# Cross-sectional study of cytomegalovirus shedding and immunological markers among seropositive children and their mothers

**DOI:** 10.1186/s12879-014-0568-2

**Published:** 2014-11-12

**Authors:** Jennifer D Stowell, Karen Mask, Minal Amin, Rebekah Clark, Denise Levis, Will Hendley, Tatiana M Lanzieri, Sheila C Dollard, Michael J Cannon

**Affiliations:** National Center on Birth Defects and Developmental Disabilities, Centers for Disease Control and Prevention, 1600 Clifton Rd NE, MS E-86 Atlanta, 30333 GA USA; National Center for Immunization and Respiratory Diseases, Centers for Disease Control and Prevention, Atlanta, GA USA; Emory University, Atlanta, GA USA

**Keywords:** Congenital, Cytomegalovirus, Transmission, Pregnancy, Children

## Abstract

**Background:**

Congenital cytomegalovirus (CMV) is the leading infectious cause of birth defects in the United States. To better understand factors that may influence CMV transmission risk, we compared viral and immunological factors in healthy children and their mothers.

**Methods:**

We screened for CMV IgG antibodies in a convenience sample of 161 children aged 0-47 months from the Atlanta, Georgia metropolitan area, along with 32 mothers of children who screened CMV-seropositive. We assessed CMV shedding via PCR using saliva collected with oral swabs (children and mothers) and urine collected from diapers using filter paper inserts (children only).

**Results:**

CMV IgG was present in 31% (50/161) of the children. Half (25/50) of seropositive children were shedding in at least one fluid. The proportion of seropositive children who shed in saliva was 100% (8/8) among the 4-12 month-olds, 64% (9/14) among 13-24 month-olds, and 40% (6/15) among 25-47 month-olds (P for trend = 0.003). Seropositive mothers had a lower proportion of saliva shedding (21% [6/29]) than children (P < 0.001). Among children who were shedding CMV, viral loads in saliva were significantly higher in younger children (P <0.001); on average, the saliva viral load of infants (i.e., <12 months) was approximately 300 times that of two year-olds (i.e., 24-35 months). Median CMV viral loads were similar in children's saliva and urine but were 10-50 times higher (P < 0.001) than the median viral load of the mothers' saliva. However, very high viral loads (> one million copies/mL) were only found in children's saliva (31% of those shedding); children's urine and mothers' saliva specimens all had fewer than 100,000 copies/mL. Low IgG avidity, a marker of primary infection, was associated with younger age (p = 0.03), higher viral loads in saliva (p = 0.02), and lower antibody titers (p = 0.005).

**Conclusions:**

Young CMV seropositive children, especially those less than one year-old may present high-risk CMV exposures to pregnant women, especially via saliva, though further research is needed to see if this finding can be generalized across racial or other demographic strata.

**Electronic supplementary material:**

The online version of this article (doi:10.1186/s12879-014-0568-2) contains supplementary material, which is available to authorized users.

## Background

Congenital cytomegalovirus (CMV) is a leading viral cause of childhood disability that remains underemphasized due to challenges in diagnosis, prevention, and treatment [1]-[5]. Many people are infected with CMV, yet most of these infections are asymptomatic and therefore unlikely to be diagnosed [6]. Thus, a pregnant woman may have or acquire CMV infection and be unaware that she is infected and at risk of passing the infection to her fetus [7]-[10]. Furthermore, because most women are unaware of CMV, and many pregnancies are unplanned (37% in the United States [11]), opportunities for preventing congenital CMV through behavioral change are often missed [1],[12]-[14].

Reducing risk for pregnant women requires a sound understanding of CMV transmission routes and sources. CMV transmission appears to require direct contact with infected body fluids. Seroconversion data point to young children as an important source of infection for pregnant women. In a comprehensive literature review, annual seroconversion rates were much higher among child care workers (8.5%) and parents with a child known to be shedding CMV (24%) than among the general population of pregnant women, healthcare workers, or parents with a child known not to be shedding CMV (all with annual seroconversion rates of ~2%) [15]. CMV infection can also result from intimate contact between adults [16],[17].

A better understanding of the relative importance of children versus adults as sources of CMV infection might lead to improved prevention messages for pregnant women. Their risk of CMV infection is influenced by the different ways in which they interact with children and adults, but other factors may also be important. Although young children are more prone to transfer body fluids to others and into their surrounding environment, adults also exchange body fluids with one another, perhaps less indiscriminately, but with some frequency nonetheless. Thus, it seems likely that infection risk may also be influenced by viral or immunological factors, or by recency of infection in the source (i.e., primary vs. non-primary infection). Although some of these factors, such as prevalence of IgG antibody positivity or viral shedding, have been explored thoroughly in children and adults, other factors, such as IgG titers, IgG avidity, viral loads, recency of infection, or specimen type (e.g., saliva vs. urine), remain understudied. In addition, most studies of these other factors [18]-[32] have not compared children and adults or have been done in congenitally infected children rather than in postnatally infected healthy children-the latter being a more important infection source because they greatly outnumber children with congenital infection.

To better understand factors that may influence CMV transmission risk for pregnant women, we carried out a descriptive epidemiology study of viral and immunological factors in healthy children and compared them to factors in adults (i.e., their mothers). In this way we hoped to inform possible behavioral measures for preventing maternal CMV infection or reinfection during pregnancy.

## Methods

### Study population

Over the course of approximately six months we enrolled a convenience sample of children without chronic medical conditions aged 0-47 months from the Atlanta, GA metropolitan area for participation in this cross-sectional study. None of the children had a diagnosis of congenital CMV infection. Aside from age, the only other inclusion requirement was that the child be using diapers. We used a variety of recruitment methods including posted flyers, e-mail list announcements, word-of-mouth, and on-site recruitment in outpatient pediatric clinics. Subsequently, we enrolled the mothers (N = 32) of those children who tested CMV-seropositive and were selected for longitudinal follow-up (Cannon et al., companion paper). The study was approved by the Institutional Review Board (IRB) at the U.S. Centers for Disease Control and Prevention (CDC). Informed consent for each participant, signed by the mother, was obtained during the enrollment process.

We administered a survey at the time of enrollment to capture demographic and socioeconomic data such as sex, age, race/ethnicity, day care attendance, household income, insurance status, and mother's educational attainment and knowledge of CMV.

### Specimen collection & testing

Children were tested for CMV IgG antibody in serum (i.e., CMV seropositivity) and CMV DNA in saliva and urine (CMV shedding). Seropositive children were enrolled in a 12-week longitudinal follow-up study described elsewhere (Cannon et al., companion paper). The mothers of CMV-seropositive children were typically enrolled 2-4 weeks after their child's visit, and were tested for CMV IgG antibody in serum and CMV DNA in saliva.

Blood specimens for antibody testing were taken via finger stick (from mothers and older children) or heel stick (from younger children). Blood was collected on a dried blood spot (DBS) card which was allowed to dry. DBS cards were delivered at room temperature to the laboratory within 24 hours of collection and were stored at -20°C pending laboratory testing. Serum was eluted from the DBS as described [33] and tested using a standard CMV ELISA assay (Sera Quest^®^, Doral, Florida).

CMV IgG titration was done on blood specimens (when enough remained) by performing two-fold dilutions and testing by ELISA. Sufficient specimen remained to obtain antibody titer results for 36 of 50 seropositive children and 28 of 29 seropositive mothers.

CMV IgG avidity testing was performed on blood (when enough remained) using standard Euroimmune^®^ ELISA kits (Luebeck, Germany) to identify recent infection. Sufficient specimen remained to obtain antibody avidity results for 40 of 50 seropositive children and 29 of 29 seropositive mothers.

Saliva specimens from children were collected using sterile oral swabs. Each swab was placed in the child's mouth for at least 20 seconds to ensure adequate absorption of saliva, and was then inserted into a collection tube. Mothers' saliva was collected by having study subjects expectorate directly into sterile tubes. All specimens were kept refrigerated during transport to the laboratory within 24 hours, where they were stored at -80°C pending laboratory testing.

Viral DNA was extracted from each swab through a quick extract method to detect CMV DNA in saliva. After thawing, 300 μl of lysis/elution buffer were added to each collection tube, which was then incubated and mixed at 56°C for one hour and then at 100°C for two minutes with agitation. The tube was then rapidly cooled on ice for 5-10 minutes, after which it was centrifuged for 1-2 minutes. The swab was removed and the specimen was tested by CMV real-time PCR, targeting the glycoprotein B gene [23].

Children's urine was collected using a diaper insert. Whatman^®^ 903 filter paper (1″ × 4″) was inserted into the inner panel of the diaper prior to placement on the child. After urination, the insert was removed from the diaper and allowed to air dry. The insert was transported to the laboratory within 24 hours where it was stored at -80°C pending laboratory testing.

Viral DNA was extracted from the filter paper via the thermal shock method [34] to detect CMV DNA in urine, with modifications as described by Kharrazi et al. [33]. Taqman-based PCR was then performed that targeted the CMV glycoprotein B gene [23].

The limits of PCR detection were estimated to be 1,600 copies/mL for saliva and 16,000 copies/mL for urine. These limits are considerably higher than our detection limit for sterile specimens (e.g., blood) collected in clinical settings, which is 70 copies/mL. Two factors led to these higher limits of detection: 1) To avoid false-positives, the PCR assay cutoff was raised five-fold from one copy per reaction (70 copies/ml) to five copies per reaction (350 copies/ml) because saliva is not sterile and urine was collected in an unsterile manner, and unsterile specimens are more susceptible to trace amounts of contamination from the environment; 2) The methods of specimen collection (i.e., swabs and filter paper) were necessary to enable in-home collection by mothers, but they resulted in reduced sample volume which raised the limit of detection approximately five-fold for saliva in swabs and 50-fold for urine in filter paper.

Notably, urine dried on filter paper was usually colorless. To confirm the presence of urine we tested for urea, following the manufacturer’s instructions (Bioassay Systems^®^, Hayward, CA), in a sample immediately adjacent to the sample used for PCR. During recruitment, we were unable to collect a urine specimen from seven of the children, and an additional three urine specimens did not have detectable urea on the filter paper and were thus excluded from analysis. Consequently, urine results were available for only 151 children.

### Statistical analysis

Statistical analyses were done using SAS version 9.3 (Cary, NC). We calculated P-values for proportions by using the Chi-Square or Fisher’s exact tests, as appropriate. When comparing viral loads or antibody titers, we used the Wilcoxon Rank-Sum test. To evaluate associations with age, we performed linear regressions on log_10_ transformed viral loads and on log_e_ transformed antibody titers.

## Results

**Figure 1:**
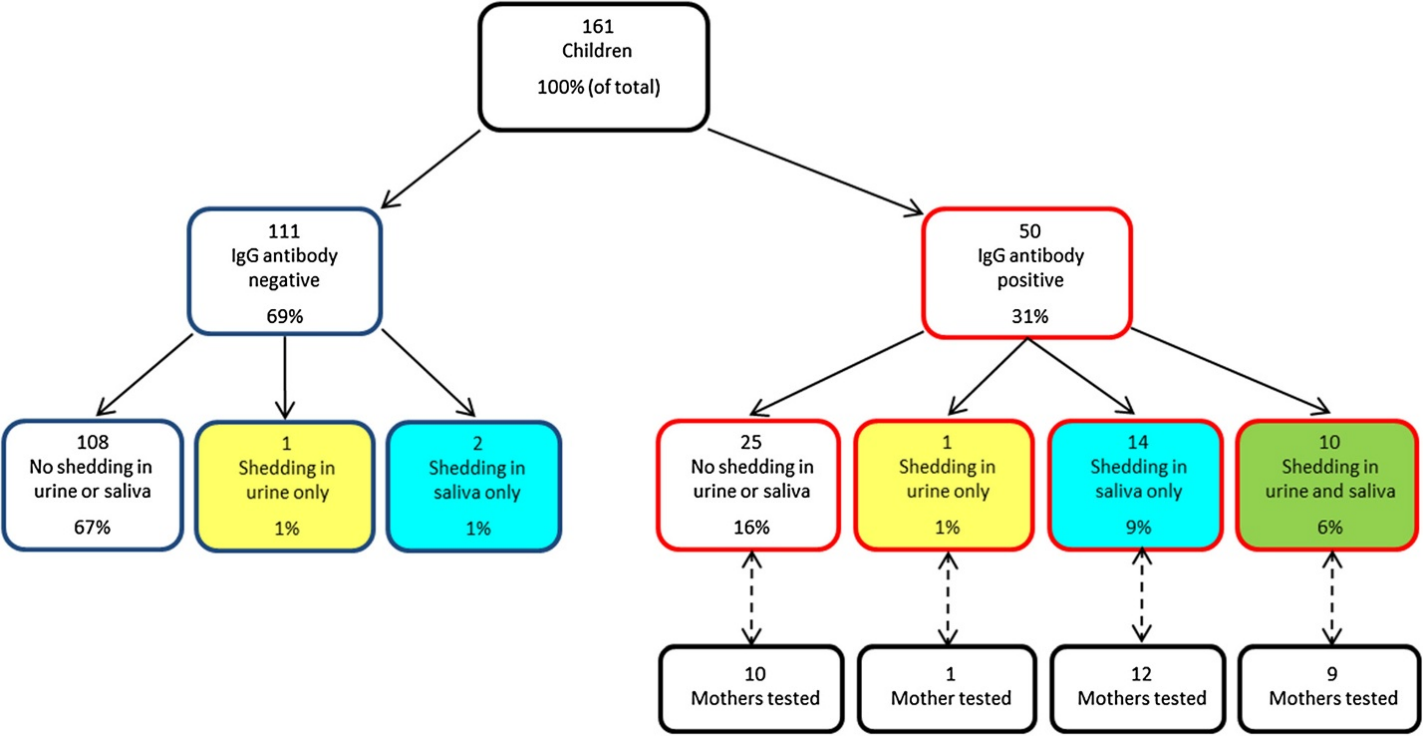
**Number, antibody status, and CMV shedding status of children enrolled in the study.** Red outlines represent children *with* CMV IgG antibody and blue outlines represent children *without* CMV IgG antibody. Yellow shading represents children shedding CMV in urine only, blue shading represents children shedding in saliva only, and green shading represents children shedding in both urine and saliva. CMV testing was also done in a subset of mothers of children who were CMV antibody positive.

We screened 161 children during the study (Table 1). The children were different from the general population of U.S. children in important ways, including that their parents had above average educational attainment and household income and were more likely to have health insurance through their employers. Nearly one third (31%) of the children were CMV-seropositive (Table 1, Figure 1). Of these, half were shedding CMV in saliva and/or urine (Figure 1). Of the 111 seronegative children, three were shedding CMV in saliva or urine (Figure 1). We also screened 32 mothers of CMV-seropositive children, of whom 29 (91%) were CMV-seropositive (Table 1). The age range of their children was similar to the age range of all the children in the study (data not shown).

**Table 1:** **Characteristics of children and mothers**

Variable	Result	Comparable U.S. averages
Sex of child		(US population <5yrs in 2010) [35]
Female	45% (72/161)	48.9%
Male	55% (89/161)	51.1%
Age of children		
0-12 months	37% (60/161)	NA
13-24 months	35% (57/161)	NA
25-47 months	27% (44/161)	NA
Maternal age	Median =34 years (range =21-46)	NA
Race*		[36]
Non-Hispanic White	69% (111/160)	79.6%
Asian/Asian American	18% (29/160)	4.6%
Black/African American	8% (13/160)	12.9%
Other	4% (7/160)	2.9%
Ethnicity		[36]
Hispanic	4% (7/161)	15.8%
Not Hispanic	96% (154/161)	84.2%
Maternal educational attainment		[37]
Professional degree	18% (29/161)	10.2%
Master's degree	22% (36/161)	
Bachelor's degree	35% (57/161)	19.4%
Some college	20% (32/161)	27.3% (includes associate degrees)
High school/GED	1% (2/161)	30.7%
Less than high school	3% (5/161)	12.4%
Median household income	$75,000-$100,000	$49,777 [38]
Insurance coverage		[39]
Employer	76% (123/161)	55.8%
Self-insured	6% (10/161)	8.2%
Public	4% (6/161)	15.7% (Medicaid stats only)
Don't know/not covered	14% (22/161)	16.7% (uninsured)
Child ever attended day care	39% (63/161)	32.9% (non-relative care) [40]
CMV seropositive- children	31% (50/161)	37.5% [41]
CMV seropositive - mothers	91% (29/32)	56.7% [41]

*Data missing for one child.

**Figure 2:**
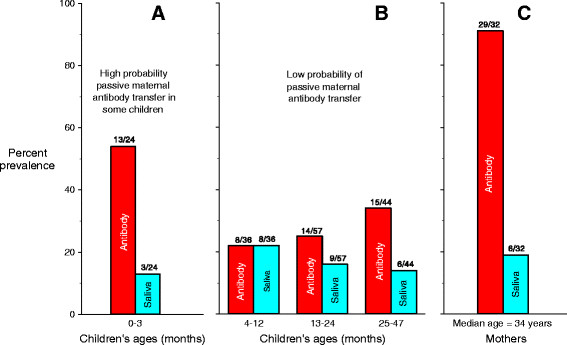
**Prevalences of CMV IgG antibody and CMV shedding in saliva among children as a function of age in months.** Prevalences of CMV IgG antibody and CMV shedding in saliva are also shown for the mothers who were screened. Red shading represents antibody results and blue shading represents saliva results. Panel **A** shows data from children ages 0-3 months; Panel **B** shows data from children ages 4-47 months; Panel **C** shows data from mothers. Antibody prevalences of mothers and children are not directly comparable because the children came from an unselected population whereas the mothers were selected for testing only if their children were CMV-seropositive, and therefore the seroprevalence among mothers was higher than would be expected in a general population.

CMV seroprevalence was higher among older children, with the exception that 0-3 month-olds had an elevated seroprevalence (Figure 2), presumably the result of some having antibodies transferred passively from their mothers. Among the 13 seropositive children aged 0-3 months, only five had evidence of infection-either viral shedding or low avidity antibodies. Among seropositive children aged 4-12 months, all were shedding CMV and therefore were likely to have had their own infection, rather than maternal antibodies only.

Among children, prevalence of shedding did not change significantly with age in either saliva (P for trend =0.70) or urine (P for trend =0.63). However, the proportion of seropositive children who shed in saliva decreased (Figure 2) from 100% (8/8) among the 4-12 month-olds, to 64% (9/14) among 13-24 month-olds, to 40% (6/15) among 25-47 month-olds (P for trend = 0.003). Seropositive mothers had an even lower proportion of saliva shedding (21% [6/29]). Of the three seronegative mothers, two had children who were shedding in saliva and/or urine, and therefore would have elevated risk of acquiring CMV from their children. None of the three seronegative mothers seroconverted during the 12-week longitudinal follow-up study (Cannon et al., companion paper).

**Figure 3:**
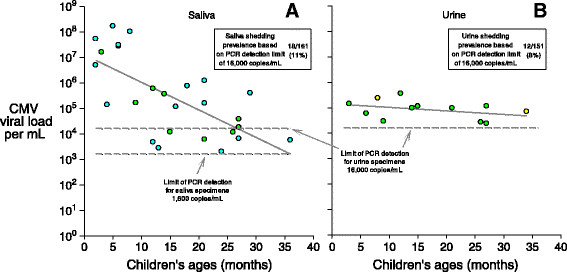
**CMV viral loads per mL as a function of Children's ages in months.** Panel **A** shows results for saliva viral loads and panel **B** shows results for urine viral loads. Circles are only plotted for children who were shedding; negative results (i.e., viral loads below the limit of detection) are not plotted. Yellow circles represent children shedding CMV in urine only, blue circles represent children shedding in saliva only, and green circles represent children shedding in both urine and saliva. The regression line in Panel **A** is log_10_ (*CMV viral load*) =7.1 - 0.108 (*age in months*), with r^2^ = 0.46 and P < 0.001; the regression line in Panel **B** is log_10_ (*CMV viral load*) =5.2 - 0.014 (*age in months*), with r^2^ = 0.14 and P = 0.22.

Although shedding prevalence was higher (Figure 1) in Children's saliva (16% [26/161]) than in urine (8% [12/151]), a direct comparison is inappropriate because the limit of PCR detection for saliva (1,600 copies/mL) was lower than for urine (16,000 copies/mL). When using the less sensitive limit for both fluids (i.e., 16,000 copies/mL), the difference between shedding prevalences (Figure 3) was small (11% vs. 8%, P =0.34).

**Figure 4:**
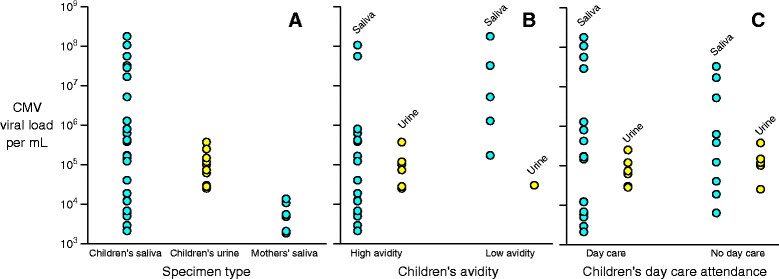
**CMV viral loads per mL stratified by three different variables.** Panel **A** shows viral loads stratified by specimen type (saliva vs. urine) and source (child vs. mother). Panel **B** shows viral loads for children only stratified by whether the child had high or intermediate versus low CMV IgG antibody avidity. Panel **C** shows viral loads stratified by day care attendance. Circles are only plotted for children who were shedding; negative results (i.e., viral loads below the limit of detection) are not plotted. Blue circles represent saliva results and yellow circles represent urine results.

Among children who were shedding CMV, viral loads were significantly lower (Figure 3) at older ages for saliva (P <0.001) but not for urine (P = 0.22). On average, the saliva viral load of infants (i.e., <12 months) was approximately 300 times that of 2 year-olds (i.e., 24-35 months) (Figure 3). Median CMV viral loads were similar in Children's saliva and urine (Figure 4A, Table 2), but were 10-50 times higher (P < 0.001) than the median viral load of the mothers' saliva (Figure 4A, Table 2). Of note, viral loads at the high end of the distributions were even more disparate-for example, more than 25% of the Children's saliva viral loads were greater than one million copies/mL, while all of the Children's urine viral loads and mothers' saliva viral loads were less than 100,000 copies/mL (Figure 4A). Among children shedders, low antibody avidity was associated with higher viral loads in saliva (Figure 4B, Table 2), but day care attendance was not associated with higher viral loads in saliva or urine (Figure 4C, Table 2).

**Table 2:** **Associations between select demographic, viral, and immunological variables**

Variable	CMV shedding in saliva and/or urine	P-value	Saliva viral load		P-value^a^	Urine viral load		P-value^b^	IgG antibody	P-value	Low IgG avidity	P-value	IgG titers^-1^(GMT)^c^	P-value
			Mean	Median		Mean	Median							
Group														
Children	17% (28/161)		1.6 × 10^7^	1.6 × 10^5^		1.1 × 10^5^	9.6 × 10^4^	0.37^d^	31% (50/161)		18% (7/40)		224	
Mothers	19% (6/32)	0.83	6.1 × 10^3^	4.9 × 10^3^	**<0.001**	-	-	**<0.001** ^**e**^	91% (29/32)	^f^	10% (3/29)	0.50	464	**0.002**
CMV IgG avidity (all)														
Low	60% (6/10)		1.6 × 10^6^	2.5 × 10^6^		-	-		-		-		136	
High or intermediate	39% (23/59)	0.30	4.4 × 10^4^	1.2 × 10^4^	**0.02**	-	-	NA	-	NA	-	NA	353	**0.005**
CMV shedders (children only)														
No	-		-	-		-	-		19% (25/133)		12% (2/17)		321	
Yes	-	NA	-	-	NA	-	-	NA	89% (25/28)	**<0.001**	23% (5/23)	0.68	290	0.68
Day care attendance (children only)														
Never	9% (9/98)		5.9 × 10^6^	3.7 × 10^5^		1.4 × 10^5^	1.1 × 10^5^		27% (26/98)		21% (4/19)		192	
Ever	30% (19/64)	**<0.001**	2.1 × 10^7^	1.6 × 10^5^	0.63	8.9 × 10^4^	6.5 × 10^4^	0.38	38% (24/63)	0.06	14% (3/21)	0.69	258	0.35

^a^Wilcoxon Rank-Sum test comparing log_10_ viral loads in saliva.

^b^Wilcoxon Rank-Sum test comparing log_10_ viral loads in urine.

^c^Wilcoxon Rank-Sum test comparing geometric mean titers (GMT).

^d^Comparison is between Children's saliva and urine.

^e^Comparison is between Children's urine and mothers' saliva.

^f^Antibody prevalences between mothers and children were not directly comparable because the children came from an unselected population whereas the mothers were selected for testing only if their children were CMV-seropositive, and therefore the seroprevalence among mothers was higher than would be expected in a general population.

Dashes are inserted where a comparison does not make sense or is redundant, or where data were insufficient.

P-values comparing proportions are chi-squared or Fisher’s exact test as appropriate; p-values less than 0.05 are highlighted in bold font.

**Figure 5:**
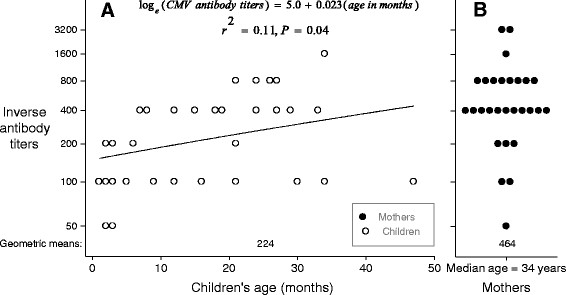
**CMV IgG antibody titers among children and mothers.** Panel **A** shows antibody titers as a function of age in months, including a regression line plotted to show the linear relationship between age in months and log_e_ viral loads. Panel **B** shows antibody titers among mothers, who had a median age of 34 years. Overall geometric mean antibody titers for children and for mothers are shown near the bottom of each panel. Circles are only plotted for individuals who had CMV IgG antibodies; negative results (i.e., antibody titers of zero) are not plotted. Black circles represent mothers' results and white circles represent Children's results.

**Figure 6:**
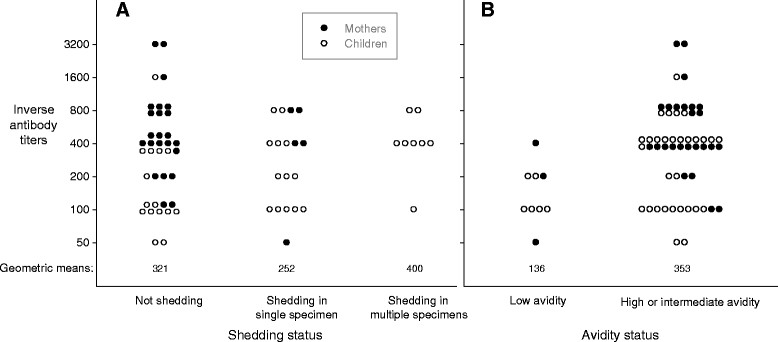
**CMV IgG antibody titers as a function of CMV shedding status and CMV IgG antibody avidity status.** Panel **A** shows antibody titers for individuals not shedding CMV, shedding CMV in only one specimen (urine or saliva), or shedding CMV in both urine and saliva. Panel **B** shows antibody titers for individuals with low avidity versus high or intermediate avidity. Geometric mean antibody titers for each category are shown near the bottom of each panel. Circles are only plotted for individuals who had CMV IgG antibodies; negative results (i.e., antibody titers of zero) are not plotted. Black circles represent mothers' results and white circles represent Children's results. For easier viewing, some of the circles are slightly offset from their true values; actual values for inverse antibody titers consisted only of the following: 50, 100, 200, 400, 800, 1600, and 3200.

NA, not applicable.

mothers' antibody titers were higher than those of children (Table 2, Figure 5B), and among children antibody titers increased with age (Figure 5A). Children 0-12 months were more likely to have low avidity antibodies (36%, 6/17) than were older children (4% [1/23]) (P = 0.03). Antibody titers were not associated with shedding in one or more specimen types (Figure 6A, all pairwise P values >0.20), but were associated with high or intermediate antibody avidity (Figure 6B, Table 2, P = 0.005). All three mothers with low antibody avidity (i.e., suggesting recent infection) had children who had high viral loads in saliva (>750,000 copies/mL), but only one of these mothers was shedding herself.

Day care attendance was associated with a three-fold higher prevalence of viral shedding (Table 2, P <0.001) and with an 11% higher seroprevalence (38% vs. 27%), though the latter association did not achieve conventional levels of statistical significance (Table 2, P = 0.06). In contrast, day care attendance was not a predictor of high CMV viral loads in saliva or urine, low avidity antibodies, or high antibody titers (Table 2).

## Discussion

Our study of the descriptive epidemiology of CMV infection among children and mothers produced several new findings that have implications for prevention of CMV infection among women of reproductive age: 1) more than half of CMV-seropositive healthy young children shed CMV DNA in saliva and/or urine, a much higher shedding rate than that of seropositive healthy adults; 2) among seropositive children, the prevalence of shedding and the magnitude of CMV viral loads tended to be greater among younger children; 3) CMV viral loads were typically higher in Children's saliva and urine than in mothers' saliva; 4) the highest viral loads (> one million copies/mL) were only found in Children's saliva; 5) low CMV IgG avidity was associated with younger age, CMV shedding, and low CMV IgG titers; and 6) high CMV IgG titers were associated with older age. We will discuss each of these new findings and their implications in turn.

Although adults have higher CMV seroprevalence than children [41], we found that seropositive children are much more likely to shed CMV. In our study, more than half of the CMV-seropositive children were shedding compared to one-fifth of the CMV-seropositive mothers. Furthermore, after excluding the children aged 0-3 months, presumably all of whom were likely to have had passively acquired maternal antibodies, we found that younger seropositive children were more likely to shed CMV than older seropositive children. The higher proportion of shedding in seropositive children, especially younger children, may help explain why they are an important source of CMV infection [15]. It also suggests that adults may have better immune control of CMV, that duration of shedding is longer for primary infections (since the children are more likely to have had recent primary infections), or that children are more likely to be exposed to reinfections.

The finding of a high proportion of shedding among seropositive children is new; although dozens of studies have measured CMV shedding among children, we could only identify eight previous studies that measured both viral shedding and antibody among healthy children (i.e., children without congenital CMV) [20],[22],[42]-[47]. However, most of these studies either did not report the shedding proportion among the seropositives or did not look at both laboratory tests in the same children. Only one of these studies [47] reported an unbiased proportion of shedding among seropositive children-33% (7/21)-but most of the children were older-in fact, only five seropositive children were under the age of five, which may, in light of our finding of less shedding among older children, have led to a lower shedding proportion in that study. Importantly, our prevalence of shedding in seropositive adults is likely to be an overestimate because, unlike the children in the study, the mothers were selected for testing because they already had a risk factor, i.e. a CMV seropositive child. This may explain why our shedding proportion (21%) was somewhat higher than that of previous studies of seropositive women, where the summary proportion of shedding in oral secretions was 13% (74/556), though the difference was not statistically significant [24],[25],[48]-[51].

In contrast to some previous findings, we found that CMV shedding in children was similar (using equivalent limits of PCR detection) for saliva (11%) and urine (8%). Previous studies that measured both urine and saliva shedding in healthy children found CMV more frequently in urine, with the prevalence difference ranging from 2%-20% [24]. However, one important difference between our study and these previous studies was that we measured shedding using PCR while the previous studies all used viral culture, which might be partially responsible for our slightly different findings.

We also found that CMV viral loads were significantly higher in Children's saliva and urine than in mothers' saliva, suggesting that children may be an especially important source of infection for pregnant women. Furthermore, the very highest viral loads (> one million copies/mL) were all in Children's saliva, suggesting that exposure to saliva may pose an even greater risk for pregnant women than exposure to urine. We were only able to identify one previous study-from The Gambia-that compared viral loads in children and mothers [22]. In that study viral loads were similar in the saliva and urine of both groups. However, we might not expect similar results since the mothers and children in Atlanta may differ in important ways, such as nutritional status or co-morbidities, from the mothers and children in The Gambia [22]. Another study, among children at day care centers in Iowa, found slightly higher viral loads in saliva than in urine, but only four children were assessed [19].

CMV viral loads were higher among younger children, suggesting that they may be an especially important source of infection for pregnant women. This finding has not been shown previously, and might be explained in part by CMV-infected younger children being more likely to have a recent primary infection-since they have had fewer months to be exposed to reinfections-and viral primary infections tend to be associated with higher viral loads [52].

Among children, low CMV avidity was associated with younger age, higher viral loads, and lower antibody titers, which is consistent with the assumption that shedding in younger children is more likely to be associated with a primary CMV infection. In addition, the higher antibody titers among older children, and the higher antibody titers among mothers compared to children, are consistent with anti-CMV immune boosting through repeated antigenic exposures, whether due to reactivations or reinfections.

Our study had several limitations. First, our study children were identified through a convenience sample rather than a representative sample of the underlying population. They included an overrepresentation of both non-Hispanic Whites and Asians/Asian-Americans. Second, we screened only 161 children. Nevertheless, our sample was relatively large compared to previous studies that assessed CMV shedding and viral load in multiple body fluids of healthy young children. Third, we collected saliva and urine using different materials (i.e., swabs vs. filter paper), and so we could not directly compare shedding prevalences or median viral loads unless we excluded the saliva results that fell below the limit of PCR detection for urine. If we had been able to collect liquid urine we might have found a higher shedding prevalence in urine, but we may have also found that the difference between median viral loads would have become significantly higher for saliva (since some lower viral load results might have been added for urine). Fourth, we do not know whether any of the children in the study were infected congenitally because none was tested before three weeks of age. However, none of the children had a clinical diagnosis of congenital CMV and all were generally healthy. Fifth, we did not usually place the specimens in viral cultures but instead used PCR to quantify CMV DNA; thus, the presence of infectious virions was not demonstrated in most cases. This approach would be expected to overestimate the number of infectious particles in specimens. For a small number of specimens we also performed viral cultures and often found evidence for infectious virions (unpublished data). Nevertheless, as with other viruses that are typically detected using PCR, such as HIV or HSV-2, the high levels of viral DNA probably indicate the presence of infectious virions in most cases. Last, because this was a cross-sectional study, children were tested at only one time point. Because CMV shedding can be sporadic, some of the CMV-seropositive children who were not shedding at their study visit may have been shedding previously or subsequently. Thus, our estimate that 17% of children were shedders underestimates the prevalence of shedders over a longer time interval (Cannon et al., companion paper).

It is likely that some of the children originally acquired their CMV infections from their mothers via breast feeding. We did not ask questions about breast feeding and did not collect or test breast milk, so we cannot be sure whether the saliva of any of the children in the study was contaminated with CMV-infected breast milk. However, such contamination is unlikely because the study procedure mandated saliva collection no sooner than one hour after breastfeeding, and the young Children's saliva viral loads were much higher than the peak viral loads typically found in breast milk [53],[54].

Taken together, our findings can be used to inform behavioral prevention messages. To decrease transmission of CMV, the American Academy of Pediatrics (AAP) has advised hand hygiene when caring for children, particularly after changing diapers [55]. Similarly, the American College of Obstetricians and Gynecologists has advised women with young children to use safe-handling techniques after handling diapers or after exposure to respiratory secretions [56]. In addition to this advice, researchers who have conducted CMV behavioral interventions have also advised women to avoid kissing young children on the mouth, to refrain from sharing food, drink, and utensils, and to cleanse toys and other objects that may be exposed to Children's body fluids [1],[12],[14],[57],[58]. Our findings suggest that although handwashing to minimize urine exposures is important (e.g., after diaper changes), behaviors that reduce saliva exposures may be even more important. Saliva appears to have higher CMV viral loads and is more likely to get into the environment through drooling, eating, pacifier use, etc. Urine, by contrast, is usually blocked by diapers. Furthermore, saliva has more opportunities to directly contact the eyes, nose and mouth of a pregnant woman (kissing, sharing food and drinks), whereas, urine contact is typically less direct (diaper-to-hand-to-eye). Because women may not recognize how frequently they come into contact with Children's saliva, it may be important to emphasize behaviors that prevent saliva from directly contacting the mouth or other mucous membranes (e.g., kissing on the mouth or sharing food/drink and utensils).

## Conclusions

Young CMV seropositive children, especially those less than one year-old may present high-risk CMV exposures to pregnant women, especially via saliva, though further research is needed to see if this finding can be generalized across racial or other demographic strata. Data on shedding among young children will be critical for developing and evaluating prevention messages and behavioral interventions in order to identify effective strategies to prevent CMV transmission.

## Authors' contributions

JDS helped design the study, coordinated and managed the data collection, participated in data analysis, and drafted the manuscript. KM collected data and participated in data analysis. MA processed specimens and carried out laboratory testing. RC collected data and participated in data analysis. DL helped design the study and participated in data collection. WH processed specimens and carried out laboratory testing. TML participated in data analysis. SCD helped design the study and supervised the laboratory testing. MJC conceived of the study, participated in its design, coordination, and data analysis, and helped draft the manuscript. All authors read the manuscript, revised it critically for important intellectual content, and approved the final manuscript.

## Authors’ original submitted files for images

Below are the links to the authors’ original submitted files for images. Authors’ original file for figure 1 Authors’ original file for figure 2 Authors’ original file for figure 3 Authors’ original file for figure 4 Authors’ original file for figure 5 Authors’ original file for figure 6
