# Supplementary figures and images for: Cross-sectional study of cytomegalovirus shedding and immunological markers among seropositive children and their mothers
Source: BMC Infect Dis. 2014 Nov 12;14:568. doi: 10.1186/s12879-014-0568-2 (PMC4236433; doi:10.1186/s12879-014-0568-2)

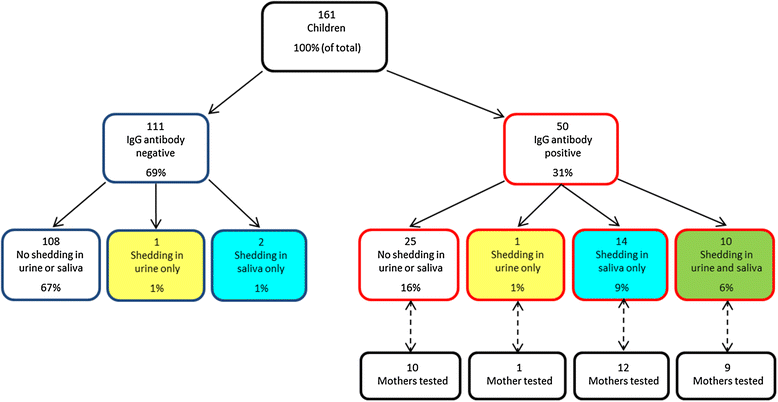

Supplement: Supplementary file 1 — Authors’ original file for figure 1 [file 12879_2014_568_MOESM1_ESM.gif]

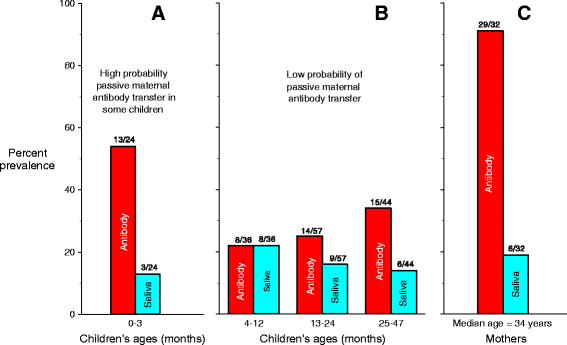

Supplement: Supplementary file 2 — Authors’ original file for figure 2 [file 12879_2014_568_MOESM2_ESM.gif]

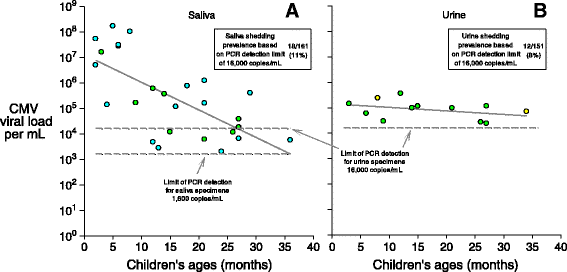

Supplement: Supplementary file 3 — Authors’ original file for figure 3 [file 12879_2014_568_MOESM3_ESM.gif]

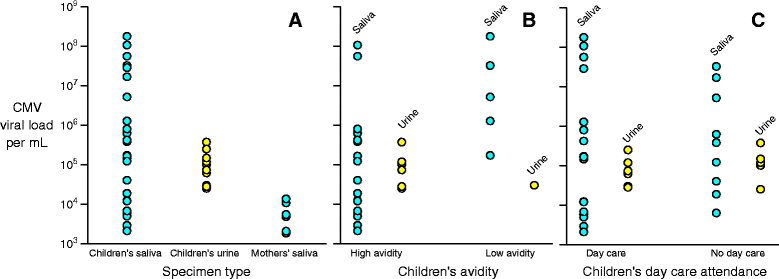

Supplement: Supplementary file 4 — Authors’ original file for figure 4 [file 12879_2014_568_MOESM4_ESM.gif]

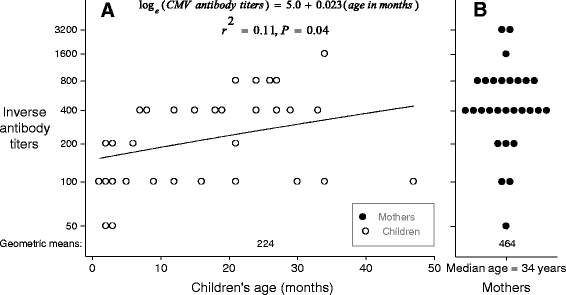

Supplement: Supplementary file 5 — Authors’ original file for figure 5 [file 12879_2014_568_MOESM5_ESM.gif]

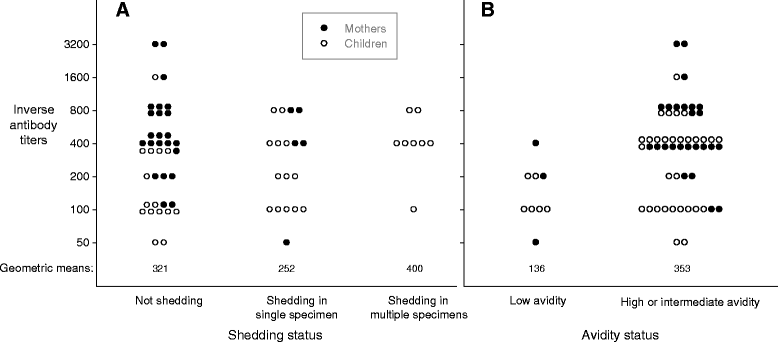

Supplement: Supplementary file 6 — Authors’ original file for figure 6 [file 12879_2014_568_MOESM6_ESM.gif]
